# Impact of alirocumab on neoatherosclerosis formation and vessel healing after drug-eluting stent implantation in patients with acute myocardial infarction: a substudy of the PACMAN-AMI trial

**DOI:** 10.1007/s10554-025-03472-0

**Published:** 2025-08-18

**Authors:** Ryota Kakizaki, Yasushi Ueki, Konstantinos C. Koskinas, Hiroki Shibutani, Sylvain Losdat, Flavio G. Biccirè, Tatsuhiko Otsuka, Jonas D. Häner, Jacob Lønborg, Christoph Kaiser, Juan F. Iglesias, Anna S. Ondracek, David Spirk, George C. M. Siontis, Joost Daemen, Thomas Engstrøm, Irene M. Lang, Lorenz Räber

**Affiliations:** 1 Department of Cardiology, Bern University Hospital, Inselspital, University of Bern, Bern, Switzerland; 2 Department of Cardiovascular Medicine, Shinshu University School of Medicine, Nagano, Japan; 3 Division of Cardiology, Department of Medicine II, Kansai Medical University, Osaka, Japan; 4 Department of Clinical Research, University of Bern, Bern, Bern, Switzerland; 5 Department of Cardiology, Itabashi Chuo Medical Center, Tokyo, Japan; 6 Department of Cardiology, Rigshospitalet, Copenhagen University Hospital, Copenhagen, Denmark; 7 Department of Cardiology, University Hospital Basel, Basel, Switzerland; 8 Department of Cardiology, Geneva University Hospitals, Geneva, Switzerland; 9 Department of Cardiology, Medical University of Vienna, Vienna, Austria; 10 Institute of Pharmacology, Bern University Hospital, University of Bern, Bern, Switzerland; 11 Department of Cardiology, Erasmus University Medical Center, Rotterdam, The Netherlands; 12 Department of Cardiology, Bern University Hospital, Inselspital, University of Bern, Freiburgstrasse 18, Bern, 3010 Switzerland

**Keywords:** Proprotein convertase subtilisin/Kexin type 9 inhibitor, Low-density lipoprotein cholesterol, Neoatherosclerosis, Neointimal hyperplasia

## Abstract

Higher on-treatment levels of low-density lipoprotein cholesterol in statin-treated patients were reportedly associated with the occurrence of neoatherosclerosis after drug-eluting stent (DES) implantation. We aimed to investigate the impact of alirocumab added to high-intensity statin therapy on neoatherosclerosis formation among patients with acute myocardial infarction (AMI) treated with DES. This was a pre-specified substudy of the PACMAN-AMI trial, a randomized, double-blind trial comparing alirocumab versus placebo added to high-intensity statin therapy in AMI patients. The present study included patients undergoing optical coherence tomography assessment of DES in the culprit lesion at one year. The frequency of neoatherosclerosis, neointimal thickness, and strut malapposition were compared between treatment groups. Among 191 patients (95 with alirocumab and 96 with placebo), neoatherosclerosis was observed in 13 patients (6.8%) at one year. There was no significant difference in the frequency of neoatherosclerosis between treatment groups (alirocumab 4.2% vs. placebo 9.4%, *P* = 0.25). Among 11 patients with lipid-laden neointima, minimal fibrous cap thickness was greater in the alirocumab group than in the placebo group (217.5 ± 122.5 vs. 87.8 ± 49.1 μm, *P* = 0.02). Neointimal thickness (136.2 ± 71.1 vs. 151.4 ± 101.4 μm, *P* = 0.45) and the frequency of malapposed struts (0.94 vs. 0.53%, *P* = 0.27) were comparable between treatment groups. Among AMI patients treated with DES, there was no significant impact of alirocumab on the frequency of neoatherosclerosis and vessel healing at one year. The observed numerical difference and the finding of more stable neoatheroma in the alirocumab group need further investigation in larger studies with extended follow-up.

**Graphical abstract:**

Alirocumab was associated with a numerically favourable, though not statistically significant, reduction in neoatherosclerosis at 1 year in AMI culprit lesions treated with newer-generation DES.

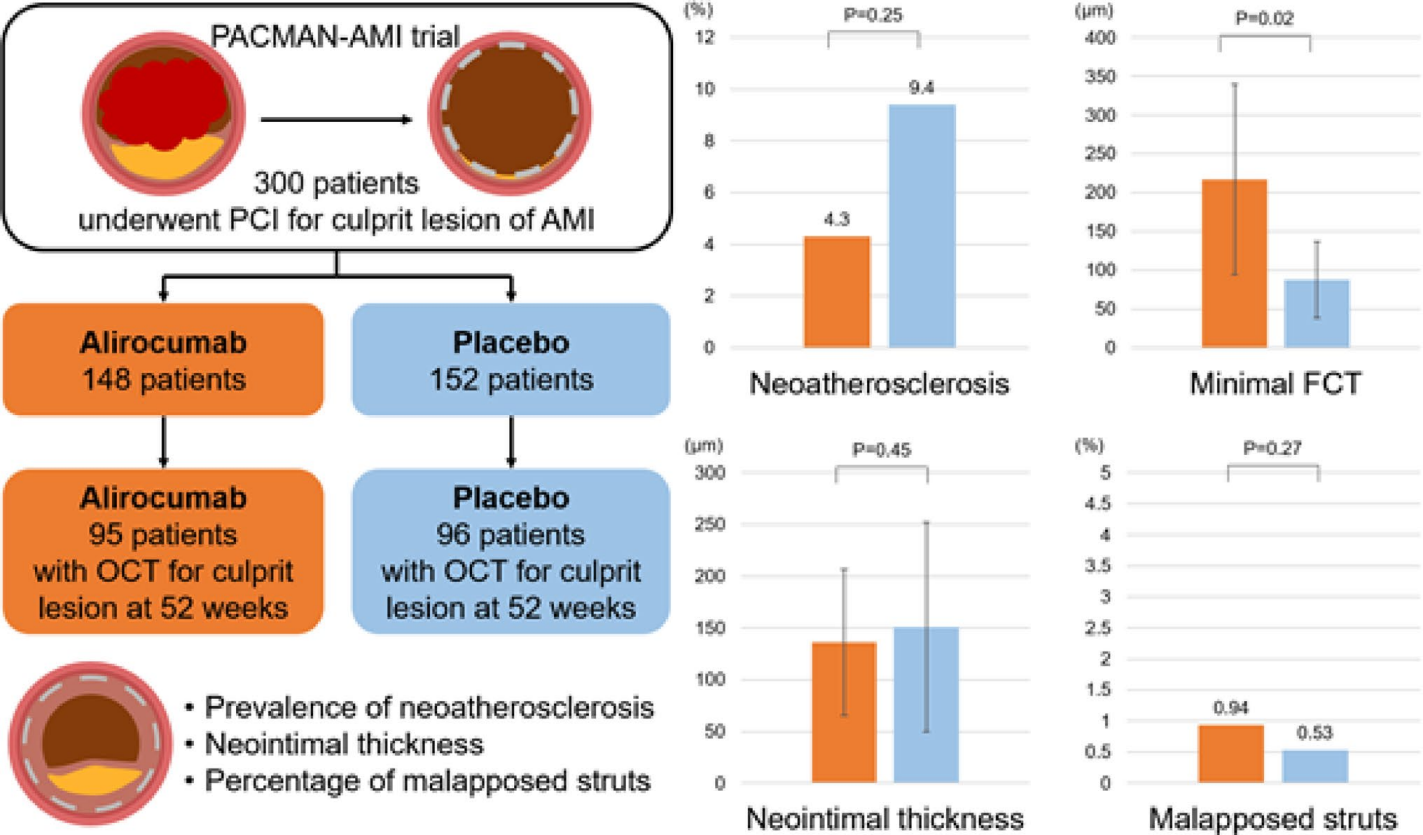

**Supplementary Information:**

The online version contains supplementary material available at 10.1007/s10554-025-03472-0.

## Introduction

Drug-eluting stents (DES) have improved the overall safety and efficacy of percutaneous coronary intervention (PCI) [1]. However, late stent failures including in-stent restenosis and stent thrombosis continue to occur at relatively low, but constant rates [2–4]. Neoatherosclerosis, histologically characterized by the accumulation of lipid-laden macrophage foam cells, necrotic core, and calcification in the neointimal tissue of the stented segment [5], is increasingly recognized as a major pathophysiological mechanism underlying late stent failures [6–8]. In culprit lesions of acute myocardial infarction (AMI), delayed vessel healing, characterized by less neointimal thickness, higher prevalence of uncovered struts, and greater inflammation, leads to more severe endothelial dysfunction and impaired endothelial barrier, potentially resulting in an accelerated formation of neoatherosclerosis [9].

Although procedure-related factors including stent type (i.e. strut thickness, polymer type), strut apposition, and underlying plaque type are important substrates for neoatherosclerosis formation and vessel healing following DES implantation, previous studies have demonstrated a significant positive association between serum low-density lipoprotein cholesterol (LDL-C) levels and the occurrence of neoatherosclerosis [10–12]. Proprotein convertase subtilisin/kexin type 9 inhibitors (PCSK9i) added to statins potently reduce LDL-C and thereby result in significant plaque regression and cardiovascular benefits compared with statin therapy alone [13, 14]. To date, the effect of intensive lipid-lowering therapy with PCSK9i on top of high-intensity statin on neoatherosclerosis formation and late stent healing remains to be elucidated.

Therefore, we have performed a predefined substudy of the randomized, double-blind PACMAN-AMI (effects of the PCSK9 antibody AliroCuMab on coronary Atherosclerosis in patieNtswith Acute Myocardial Infarction) trial to assess whether intensive lipid-lowering therapy with alirocumab impacts on the frequency of neoatherosclerosis and vessel healing.

## Methods

### Study population

The PACMAN-AMI trial (NCT03067844) was an investigator-initiated, multicenter, randomized, double-blind clinical trial conducted at 9 centers in 4 European countries (Switzerland, Austria, Denmark, and the Netherlands). The study design and main results of the PACMAN-AMI trial has been reported previously [14, 15]. In brief, the PACMAN-AMI trial included 300 patients with 18 years or older who underwent PCI of the culprit lesion for treatment of ST-elevation or non-ST-elevation myocardial infarction. Patients were randomized to receive biweekly alirocumab or placebo for 52 weeks in addition to high-intensity statin therapy (rosuvastatin 20 mg). The present pre-specified substudy included patients who underwent optical coherence tomography (OCT) imaging for the culprit lesion of AMI at 52 weeks following primary PCI. OCT recording was performed if the operator deemed it feasible for the culprit lesion at one year follow-up. PCI was performed in accordance with the European Society of Cardiology Guidelines [16] and DES selection was at the discretion of the operator. All patients provided written informed consent, and the study was approved by the ethical committee at each site.

### Acquisition and analysis of intracoronary imaging

The OCT imaging was performed using a frequency-domain OCT system (Dragon Fly, LightLab, St. Jude Medical, St. Paul, MN, USA). OCT images were analyzed at independent core laboratory (Bern University Hospital, Bern, Switzerland) by experienced analysts. Cross-sectional OCT images were evaluated quantitatively and qualitatively with an interval of 0.4 mm within the stented segments using proprietary software (QCU-CMS version 4.69 software, LKEB, Leiden, The Netherlands). Neointima was defined as the tissue between the luminal border and the endoluminal border of the struts. Neoatherosclerosis was defined as either the presence of lipid-laden neointima or calcification with a longitudinal extension of ≥ 1.2 mm, the presence of macrophage, or the presence of cholesterol crystals [17]. Fibroatheroma were characterized as a signal-poor region displaying high attenuation (to differentiate from layered neointima) with diffuse borders and a lateral extension of at least one quadrant [18]. Calcification were defined as signal-poor regions with low attenuation and clear borderlines. Macrophage was defined as lines or dots with strong signal attenuation producing a shadow with a sharply delineated lateral border. In-stent cholesterol crystal was defined as thin and linear structures with high backscattering without attenuation within the neointima. Stent strut coverage and malapposition were defined and measured as described previously [18].

### Study endpoints

Primary endpoint was the frequency of neoatherosclerosis at 1 year, and key secondary endpoints were neointimal area, neointimal thickness, and percentage of uncovered and malapposed struts at 1 year.

### Statistical analysis

Continuous variables were summarized as mean (standard deviation) or median [interquartile range]. Between-group comparisons were conducted using Student t-tests or Wilcoxon-Mann-Whitney tests, as appropriate. Categorical variables were summarized as counts (percentage) and compared between groups using Fisher’s exact tests. Statistical significance was set to 0.05. All statistical analyses were performed using Stata 17 (StataCorp LLC, College Station, TX) and R 4.4.1 (R Core Team).

## Results

### Baseline and procedural characteristics

A total of 191 patients (95 in the alirocumab group and 96 in the placebo group) undergoing OCT imaging for the culprit stents at 1 year were analyzed for the current sub-study (Supplementary Fig. 1). Baseline clinical and procedural characteristics were well-balanced between treatment groups (Tables 1 and 2). LDL-C level at follow-up was 23.2 ± 23.2 mg/dL in the alirocumab group and 73.5 ± 30.9 mg/dL in the placebo group. Among 265 patients who received serial angiography, quantitative coronary angiography results were comparable between the alirocumab group and the placebo group (Supplementary Table 1). There were no significant differences in baseline characteristics between patients with and without OCT for the culprit lesion at 1 year follow-up, except for the frequency of dyslipidemia and P2Y12 inhibitor at baseline (Supplementary Table 2). Quantitative coronary angiography results were also comparable between patients with and without OCT at 1 year follow-up.

**Table 1:** Patient characteristics and medication

	Overall *n* = 191	Alirocumab *n* = 95	Placebo *n* = 96	*P* value
Age, years	58.1 (9.5)	57.9 (10.1)	58.4 (8.9)	0.69
Men, n (%)	160 (83.8)	83 (87.4)	77 (80.2)	0.24
BMI, kg/m2	27.9 (4.3)	27.3 (3.9)	28.4 (4.6	0.06
Medical history, n (%)				
Arterial hypertension	81 (42.4)	34 (35.8)	47 (49.0)	0.08
Dyslipidemia	162 (84.8)	79 (83.2)	83 (86.5)	0.55
Diabetes	16 (8.4)	5 (5.3)	11 (11.5)	0.19
Current smoking	87 (45.5)	49 (51.6)	38 (39.6)	0.11
Previous myocardial infarction	2 (1.0)	1 (1.1)	1 (1.0)	1.00
Previous PCI	3 (1.6)	1 (1.1)	2 (2.1)	1.00
Peripheral arterial disease	3 (1.6)	1 (1.1)	2 (2.1)	1.00
Family history of CAD	59 (30.9)	28 (29.5)	31 (32.3)	0.75
Type of acute myocardial infarction, n (%)				0.47
NSTEMI	85 (44.5)	45 (47.4)	40 (41.7)	
STEMI	106 (55.5)	50 (52.6)	56 (58.3)	
Peak CK, IU/L	687 (1061)	770 (1191)	605 (915)	0.29
Peak hs-cTnT, ng/mL	1189 (2734)	1417 (3311)	961 (1991)	0.25
LVEF, %	53 (11)	53 (11)	53 (11)	0.92
Medication, n (%)				
Statin	26 (13.6)	11 (11.6)	15 (15.6)	0.53
High-intensity statin therapy	13 (6.8)	6 (6.3)	7 (7.3)	1.00
Ezetimibe	0 (0.0)	0 (0.0)	0 (0.0)	
Antiplatelet therapy				
Aspirin	13 (6.8)	7 (7.4)	6 (6.2)	0.78
P2Y12 inhibitor	3 (1.6)	0 (0.0)	3 (3.1)	0.25
Anti-coagulant	2 (1.0)	1 (1.1)	1 (1.0)	1.00
β-Blocker	15 (7.9)	5 (5.3)	10 (10.4)	0.28
ACEI	13 (6.8)	5 (5.3)	8 (8.3)	0.57
ARB	20 (10.5)	7 (7.4)	13 (13.5)	0.24

Values are count (percentage) or mean (SD). Abbreviations: ACEI: angiotensin converting enzyme inhibitor, ARB: angiotensin receptor blocker, BMI: body mass index, CAD: coronary artery disease, CK: creatinine kinase, cTnT: cardiac troponin T, LVEF: left ventricular ejection fraction, NSTEMI: non-ST-segment elevation myocardial infarction, PCI: percutaneous coronary intervention, STEMI: ST-segment elevation myocardial infarction

**Table 2:** Lesion and procedure characteristics

	Overall *n* = 191	Alirocumab *n* = 95	Placebo *n* = 96	*P* value
Target vessel location, n (%)				0.20
Left anterior descending	93 (48.7)	47 (49.5)	46 (47.9)	
Left circumflex	42 (22.0)	25 (26.3)	17 (17.7)	
Right coronary artery	56 (29.3)	23 (24.2)	33 (34.4)	
Final TIMI flow, n (%)				0.50
0	0 (0.0)	0 (0.0)	0 (0.0)	
1	0 (0.0)	0 (0.0)	0 (0.0)	
2	1 (0.5)	1 (1.1)	0 (0.0)	
3	190 (99.5)	94 (98.9)	96 (100.0)	
Number of stents, n (%)	1.0 [0.0]	1.0 [0.0]	1.0 [0.0]	0.72
Total stent length, mm	28.0 [20.0]	26.0 [20.0]	28.0 [17.0]	0.36
Stent diameter, mm	3.5 [1.0]	3.5 [1.0]	3.5 [1.0]	0.60
Polymer type, n (%)				0.88
Biodegradable polymer	98 (51.3)	49 (51.6)	49 (51.0)	
Durable polymer	89 (46.6)	43 (45.3)	46 (47.9)	
Post-dilatation, n (%)	160 (83.8%)	78 (82.1%)	82 (85.4%)	0.56
Bifurcation lesion, n (%)	30 (15.7)	12 (12.6)	18 (18.8)	0.32
Multivessel PCI, n (%)	19 (9.9)	13 (13.7)	6 (6.2)	0.10
GP IIb/IIIa inhibitors, n (%)	33 (17.3)	16 (16.8)	17 (17.7)	1.00

Values are count (percentage), mean (SD), or median [interquartile range]. Multivessel treatment and GP IIb/IIIa are at patient level. All other variables refer to the culprit lesion. Abbreviations: GP: glycoprotein, PCI: percutaneous coronary intervention, TIMI: thrombolysis in myocardial infarction

### OCT findings

**Fig. 1:**
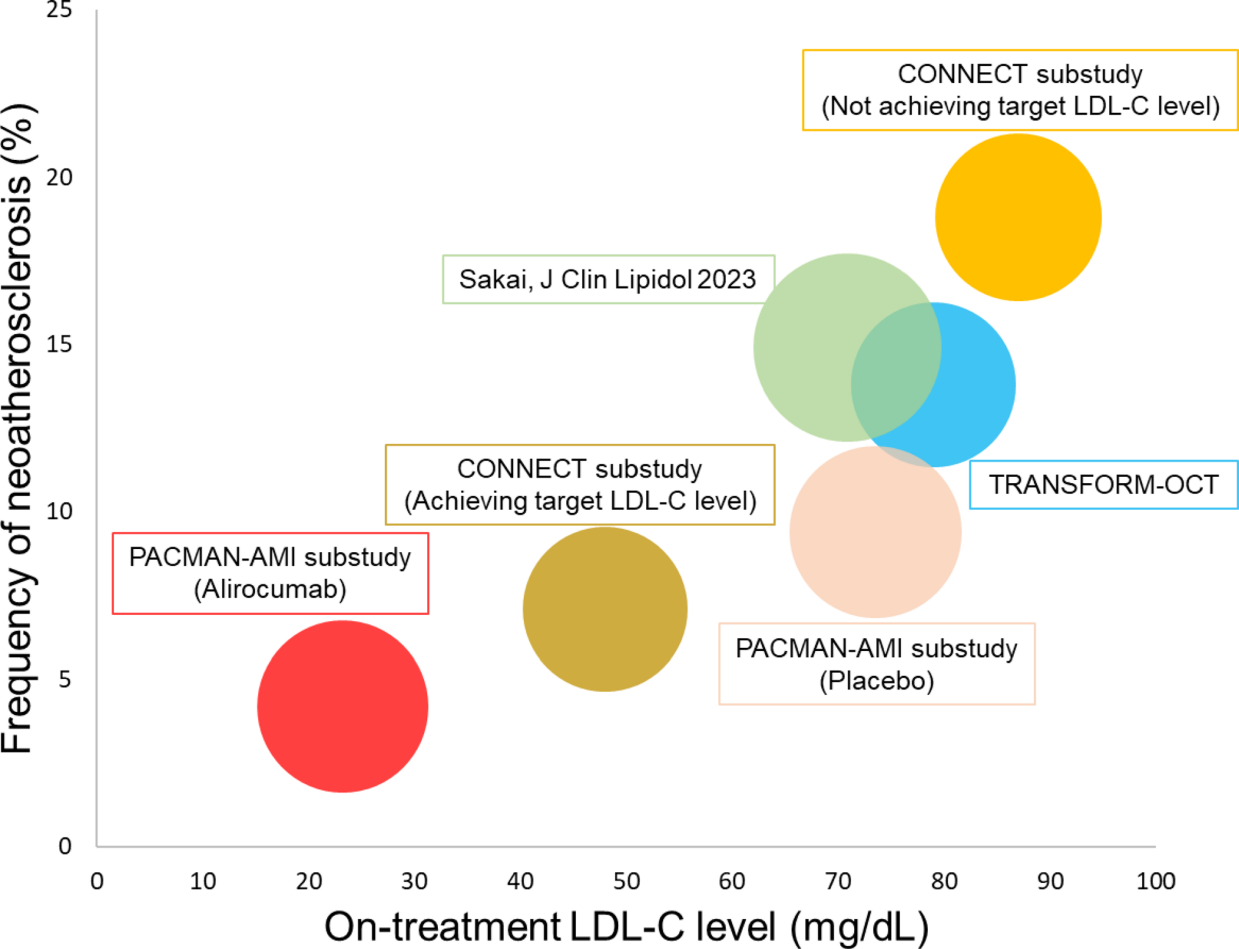
Frequency of neoatherosclerosis according to mean LDL-C levels in prospective studies. Bubble plot showing the frequency of neoatherosclerosis (y-axis) in comparison to mean on-treatment LDL-C level (x-axis). Bubble size is proportional to the overall number of patients (PACMAN-AMI alirocumab group: 95, PACMAN-AMI Placebo group: 96, CONNECT patients achieving target LDL-C level: 98, CONNECT patients not achieving target LDL-C level: 80, TRANSFORM-OCT: 87, Sakai_J Clin Lipidol 2023: 114). BP: biodegradable polymer, DP: durable polymer, EES: everolimus-eluting stents, LDL-C: low density lipoprotein cholesterol, ZES: zotarolimus-eluting stents

OCT findings are shown in Table 3. Neoatherosclerosis was observed in 13 (6.8%) patients, 4 (4.2%) in the alirocumab group and 9 (9.4%) in the placebo group (*p* = 0.25). There was no difference in the distribution of polymer type of DES between patients with and without neoatherosclerosis (biodegradable polymer: 7.1% vs. 92.9%; durable polymer: 6.7% vs. 93.3%, *p* = 1.00). The frequency of fibroatheroma, fibrocalcific plaque, macrophage, and cholesterol crystal was 5.8%, 1.0%, 1.0%, and 2.1%, respectively, without statistical differences between treatment groups. Among 11 patients with lipid-laden neointima, fibrous cap thickness was significantly greater in the alirocumab group than in the placebo group (217 ± 123 μm vs. 88 ± 49 μm, *p* = 0.02). The characteristics of patients with neoatherosclerosis were summarized in Supplementary Table 3. Biochemical measures and intracoronary imaging findings in patients with and without neoatherosclerosis were shown in Supplementary Table 4. Lp(a) levels at baseline (29.9 ± 39.3 mg/dL vs. 31.1 ± 39.8 mg/dL, *p* = 0.97) and follow-up (36.4 ± 46.6 mg/dL vs. 33.5 ± 45.5 mg/dL, *p* = 0.97) were comparable between patients with and without neoatherosclerosis. Absolute change in serum LDL-C level was − 106.5 (−135.4 to −77.5) vs. −104.3 (−111.0 to −97.6) mg/dL in patients with vs. without neoatherosclerosis. Figure 1 shows the frequency of neoatherosclerosis according to mean LDL-C levels in prospective studies.

**Table 3:** OCT analysis at 1 year

	Overall *n* = 191	Alirocumab *n* = 95	Placebo *n* = 96	*P* value
Duration from implantation, day	376 (15)	377 (14)	374 (16)	0.16
Neoatherosclerosis, n (%)	13 (6.8)	4 (4.2)	9 (9.4)	0.25
Fibroatheroma, n (%)	11 (5.8)	4 (4.2)	7 (7.3)	0.54
*Total length of fibroatheroma, mm	5.78 (4.91)	4.00 (3.35)	6.80 (5.59)	0.32
*Maximum lipid arc, degree	173 (73)	155 (38)	184 (89)	0.79
*Minimum cap thickness, µm	135 (101)	217 (123)	88 (49)	**0.02**
Fibrocalcified, n (%)	2 (1.0)	0 (0.0)	2 (2.1)	0.50
Macrophage accumulations, n (%)	2 (1.0)	1 (1.1)	1 (1.0)	1.00
In-stent cholesterol crystal, n (%)	4 (2.1)	1 (1.1)	3 (3.1)	0.62
Other findings, n (%)				
Thrombus	2 (1.0)	2 (2.1)	0 (0.0)	0.50
Microvessel	76 (39.8)	39 (41.1)	37 (38.5)	0.88
Lesion level analysis				
Analyzed stent length, mm	31.1 (14.1)	30.1 (13.6)	32.0 (14.6)	0.45
Minimal lumen area, mm^2^	4.82 (2.21)	5.09 (2.32)	4.55 (2.07)	0.09
Minimal stent area, mm^2^	6.05 (2.45)	6.21 (2.39)	5.90 (2.51)	0.37
Cross section level analysis				
Analyzed cross section per lesion, n (%)	78 (35)	77 (35)	80 (36)	0.53
Mean lumen area, mm^2^	6.98 (2.82)	7.13 (2.71)	6.83 (2.93)	0.28
Mean stent area, mm^2^	8.06 (2.97)	8.12 (2.69)	8.00 (3.23)	0.57
Neointimal area, mm^2^	1.14 (0.85)	1.07 (0.73)	1.20 (0.96)	0.60
Malapposed area, mm^2^	0.05 (0.20)	0.07 (0.25)	0.03 (0.12)	0.40
Strut level analysis				
Analyzed struts per lesion, n (%)	762 (364)	744 (353)	781 (375)	0.48
Mean neointimal thickness, µm	145 (88)	136 (71)	151 (101)	0.45
Rate of uncovered struts, %	2.7 (3.9)	3.1 (4.5)	2.3 (3.3)	0.45
Rate of malapposed struts, %	0.7 (2.4)	0.9 (2.8)	0.5 (2.1)	0.27
Incomplete apposition distance, µm	3.66 (12.04)	4.73 (13.93)	2.61 (9.77)	0.49

Values are count (percentage) or mean (SD). Abbreviations: OCT: optical coherence tomography. *calculated across 11 patients

At a cross sectional level, there were no differences in the frequency of cross sections with uncovered struts (15.0 vs. 12.13%, *p* = 0.57) and malapposed struts (4.0 vs. 1.7%, *p* = 0.24), and neointimal area (1.07 ± 0.73 vs. 1.20 ± 0.96 mm^2^, *p* = 0.60) between the two groups (Table 3). Supplementary Fig. 2 shows the correlation between neointimal area and absolute change in LDL-C, and Supplementary Fig. 3 shows the correlation between percentage of uncovered struts and absolute change in LDL-C. At a strut level, the percentage of uncovered struts (3.1 vs. 2.3%, *p* = 0.45), the percentage of malapposed struts (0.9 vs. 0.5%, *p* = 0.27) and neointimal thickness (136 vs. 151 μm, *p* = 0.45) were comparable between two groups (Table 3). Patients with neoatherosclerosis had greater neointimal area (2.11 ± 1.08 vs. 1.06 ± 0.79 mm^2^) and greater neointimal thickness (241 ± 97 vs. 137 ± 83 μm) compared with those without (Supplementary Table 5).

### Clinical outcomes

Supplementary Table 6 shows the incidence of the revascularization at 1 year. Revascularization was performed in 35 patients (18.3%). Among them, 11 patients (5.8%) underwent ischemia-driven target lesion revascularization, 2 (15.4%) with neoatherosclerosis and 9 (5.1%) without neoatherosclerosis. Ischemia-driven non-target lesion revascularization was performed in 19 patients (9.9%), 1 (7.7%) in patients with neoatherosclerosis and 18 (10.1%) in patients without neoatherosclerosis.

## Discussion

This is the first study to investigate the effect of very low LDL-C level achieved by alirocumab on neoatherosclerosis formation and vessel healing among AMI patients undergoing PCI with current generation DES. The main findings of the present study, based on OCT performed in the stented culprit lesion 1 year after primary PCI, are: (1) there were no significant differences in the frequency of neoatherosclerosis between the alirocumab and placebo groups in the context of a background high-intensity statin treatment in both treatment arms (rosuvastatin 20 mg). However, neoatheroma in the alirocumab group was more stable as suggested by a significantly greater minimal fibrous cap thickness, (2) the degree of vessel healing represented by neointimal growth, strut coverage, and strut apposition did not significantly differ between the treatment groups at 1 year following primary PCI.

### Frequency of neoatherosclerosis

Although there are several relevant differences in pathogenic mechanisms between in-stent neoatherosclerosis and native atherosclerosis, lipid diffusion and inflammatory cell migration through the endothelium are considered common pathways of atherosclerosis formation for both disease entities. Previous retrospective observational studies have demonstrated a significant correlation between the serum LDL-C level and the frequency of neoatherosclerosis [10–12, 19, 20]. The substudy of the CONNECT trial also demonstrated that neoatherosclerosis was less frequent in STEMI patients who achieved guideline-endorsed LDL-C levels and received high-intensity statin therapy 3 years after implantation of current generation DES [21]. In the current study, the frequency of neoatherosclerosis was numerically lower in the alirocumab group (4.2% vs. 9.4%), but there was no significant difference between groups despite very low LDL-C levels achieved in the alirocumab group (mean 23.2 mg/dL vs. 73.5 mg/dL). A potential explanation may be the lack of power (i.e. type II error) in view of only 13 patients with neoatherosclerosis at 1 year. The relatively short follow-up period of 1 year in our pre-specified sub-study may at least partly contribute to small number of neoatherosclerosis formation. Another explanation may be the initiation of high-intensity statin therapy (rosuvastatin 20 mg) in both groups from the first day after primary PCI, which was not the case in previous studies. Statins not only decrease LDL-C level but also reduce inflammation, which can lead to endothelial cell activation and dysfunction [22] and thus result in neoatherosclerosis formation. The level of high sensitive C-reactive protein (hsCRP) was reportedly associated with the accumulation of lipids within the neointima [10]. Unlike statins, PSCK9i have little no effect on inflammation [23] and accordingly, the on-treatment hsCRP level was similar in both treatment groups in our study (2.0 vs. 2.5 mg/L, *p* = 0.71). Interestingly, neoatheroma in the alirocumab group had a significantly greater minimal fibrous cap thickness. This finding may suggest that intensive lipid-lowering afforded by alirocumab resulted in more stable neoatheroma, along the lines of the effects of alirocumab observed in the main study investigating native atherosclerosis [24].

The frequency of neoatherosclerosis in the alirocumab group in the current study (4.2%) appeared to be among the lowest ever reported compared with previous prospective observational studies that typically found neoatherosclerosis in 11.4–15.9% between 1 and 3 years with on-treatment LDL-C levels between 63 and 80 mg/dL [17, 25, 26] (Fig. 1). Moreover, the frequency of neoatherosclerosis in the placebo group of the present study (9.4%) was also numerically lower compared to that reported in previous studies despite a similar on-treatment LDL-C level. The lower frequency in the current study may be explained by the routine use of high-intensity statin therapy, stent types, and clinical presentation. In particular, a previous pathological study reported that neointimal thickness in culprit site of AMI was significantly smaller than in coronary lesions in stable patients [9]. Given the pathological role of in-stent neointima as a basis for neoatherosclerosis formation, the lower frequency of neoatherosclerosis might be attributed to smaller neointimal tissue in the AMI culprit lesions in the current study.

Interestingly, we noted some patients with neoatherosclerosis formation within 12 months with very low on treatment LDL-C values (Supplementary Table 3), i.e. 3 patients with < 20 mg/dL. This underlines that on-treatment LDL-C should not be considered as an isolated driver of neoatherosclerosis. In these patients, hsCRP levels at follow-up were 0, 0, and 0.8 mg/L, respectively. These three patients suggest that other, incompletely understood mechanisms unrelated to LDL-C and inflammation may come into play.

### Neointimal growth and strut malapposition

In the current study, there was no significant difference in the degree of neointimal hyperplasia, strut coverage, and strut apposition. Although there have been potential concerns of late-acquired malapposition due to pronounced plaque regression induced by intensive LDL-C lowering with alirocumab, reassuringly, we did not observe such a phenomenon in the current analysis showing a low and similar frequency of malapposed and uncovered struts in both treatment groups and no correlation with on treatment LDL-C. This represents a reassuring finding with respect to the safety of DES in patients receiving intensive lipid lowering therapy.

In contrast to our findings, a previous observational OCT study in South Korea investigating the effect of on treatment LDL-C level on neointimal hyperplasia among 218 patients treated with DES has demonstrated that neointimal tissue growth from 6 to 18 months was more suppressed in patients with a lower LDL-C level (58 ± 11 mg/dL) compared with those with a higher LDL-C level (92 ± 18 mg/dL) (change in neointimal area: 0.2 vs. 0.4mm^2^, *p* = 0.01) [27]. The potential reasons for inconsistent results include the observational versus randomized controlled design and the study duration.

### Clinical implication

Neoatherosclerosis is a predominant mechanism underlying late stent failure contributing to at least 30–40% of failure cases occurring beyond the first year after revascularization. Moreover, previous studies have demonstrated its prognostic significance for adverse cardiovascular events, including cardiac death and target lesion revascularization [10, 28]. Although Lp(a) levels were previously associated with the occurrence of neoatherosclerosis in one observational study [29], we did not find such an association. More and larger studies are needed that correlate Lp(a) baseline levels with neoatherosclerosis formation. Several clinical trials investigating the effect of Lp(a) lowering on cardiovascular outcomes are currently ongoing and depending on the results, future studies should also focus on the prevention of neoatherosclerosis. The effects of PCSK9 inhibitors on stent-related outcomes remain underexplored. In a substudy of the FOURIER trial involving patients with prior PCI and followed for 2.2 years, there were significantly less revascularizations of de novo lesions, however, no significant difference was observed between evolocumab and placebo treated groups regarding the incidence of PCI for in-stent restenosis (2.0 vs. 2.3%, HR 0.84 (0.69–1.03) or stent thrombosis (0.4 vs. 0.5%, HR 0.81 (0.52–1.25). One limitation of this study is that neoatherosclerosis-related revascularizations usually occur far beyond the first year after stent implantation [30]. Further studies are warranted to elucidate the impact of intensive lipid-lowering therapy with PCSK9 inhibitors on stent-related outcomes.

### Limitations

The present study has several limitations. First, the lack of significant differences between groups may be attributable to the lack of power (i.e., type II error) due to small sample size and low frequency of neoatherosclerosis. Second, OCT imaging for the culprit stents was not performed in all patients enrolled into the PACMAN-AMI trial, which may lead to patient selection bias. However, no differences in key baseline patient, lesion and procedural characteristics between patients with and without OCT imaging at 1 year were observed (Supplementary Table 2). Third, the follow-up period of 1 year was relatively short to evaluate the occurrence of neoatherosclerosis, which can emerge consistently after 1 year and is the primary mechanism of very late (> 1 year) stent failure. Fourth, OCT findings at baseline including plaque morphology of the culprit lesion and acute stent results (i.e. stent expansion, strut apposition), known predictors of stent healing and neoatherosclerosis formation, were not available in the current study [19]. Although patients were randomly assigned to the alirocumab group and the placebo group, the potential influence of these confounding factors on the frequency of neoatherosclerosis cannot be excluded. Fifth, although all patients were treated with current generation DES in the current study, the choice of stent type was left at the operator’s discretion. Stent type was reported in Supplementary Table 7. Previous studies have reported that stent type was significantly associated with the occurrence of neoatherosclerosis [10, 31–33]. Sixth, despite its high spatial resolution, OCT cannot reliably distinguish healthy neointima from fibrin and thrombus [34].

## Conclusion

Among AMI patients undergoing PCI of the culprit lesion with newer generation DES, there was no significant impact of alirocumab on the frequency of neoatherosclerosis and vessel healing at 1 year. The observed numerical difference and the finding of more stable neoatheroma in favor of alirocumab require further investigation in larger studies with extended follow-up.

## Supplementary Information

Below is the link to the electronic supplementary material.

Supplementary Material 1

## Data Availability

The data set will be available from the corresponding author on reasonable request.

## References

[1] Piccolo R, Bonaa KH, Efthimiou O, Varenne O, Baldo A et al (2019) Drug-eluting or bare-metal stents for percutaneous coronary intervention: a systematic review and individual patient data meta-analysis of randomised clinical trials. Lancet Jun 22(10190):2503–2510. 10.1016/S0140-6736(19)30474-X 10.1016/S0140-6736(19)30474-X 31056295

[2] Lee JM, Park KW, Han JK, Yang HM, Kang HJ et al (2014) Three-year patient-related and stent-related outcomes of second-generation everolimus-eluting Xience V stents versus zotarolimus-eluting resolute stents in real-world practice (from the multicenter prospective EXCELLENT and RESOLUTE-Korea Registries). Am J Cardiol Nov 1(9):1329–1338. 10.1016/j.amjcard.2014.07.065 10.1016/j.amjcard.2014.07.065 25217457

[3] Camenzind E, Wijns W, Mauri L, Kurowski V, Parikh K et al (2012) Stent thrombosis and major clinical events at 3 years after zotarolimus-eluting or sirolimus-eluting coronary stent implantation: a randomised, multicentre, open-label, controlled trial. Lancet Oct 20(9851):1396–1405. 10.1016/S0140-6736(12)61336-1 10.1016/S0140-6736(12)61336-1 22951082

[4] Zanchin C, Ueki Y, Zanchin T, Haner J, Otsuka T et al (2019) Everolimus-Eluting biodegradable polymer versus Everolimus-Eluting durable polymer stent for coronary revascularization in routine clinical practice. JACC Cardiovasc Interv Sep 9(17):1665–1675. 10.1016/j.jcin.2019.04.046 10.1016/j.jcin.2019.04.046 31422088

[5] Takano M, Yamamoto M, Inami S, Murakami D, Ohba T et al (2009) Appearance of lipid-laden intima and neovascularization after implantation of bare-metal stents extended late-phase observation by intracoronary optical coherence tomography. J Am Coll Cardiol Dec 29(1):26–32. 10.1016/j.jacc.2009.08.032 10.1016/j.jacc.2009.08.032 20117359

[6] Taniwaki M, Radu MD, Zaugg S, Amabile N, Garcia-Garcia HM et al (2016) Mechanisms of very late Drug-Eluting stent thrombosis assessed by optical coherence tomography. Circulation Feb 16(7):650–660. 10.1161/CIRCULATIONAHA.115.019071 10.1161/CIRCULATIONAHA.115.019071 26762519

[7] Nakamura D, Attizzani GF, Toma C, Sheth T, Wang W et al (2016) Failure mechanisms and neoatherosclerosis patterns in very late Drug-Eluting and Bare-Metal stent thrombosis. Circ Cardiovasc Interv Sep 9(9). 10.1161/CIRCINTERVENTIONS.116.003785 10.1161/CIRCINTERVENTIONS.116.003785 27582113

[8] Kawai K, Virmani R, Finn AV (2022) In-Stent restenosis. Interv Cardiol Clin Oct 11(4):429–443. 10.1016/j.iccl.2022.02.005 10.1016/j.iccl.2022.02.005 36243488

[9] Nakazawa G, Finn AV, Joner M, Ladich E, Kutys R et al (2008) Delayed arterial healing and increased late stent thrombosis at culprit sites after drug-eluting stent placement for acute myocardial infarction patients: an autopsy study. Circulation Sep 9(11):1138–1145. 10.1161/CIRCULATIONAHA.107.762047 10.1161/CIRCULATIONAHA.107.762047 18725485

[10] Kuroda M, Otake H, Shinke T, Takaya T, Nakagawa M et al (2016) The impact of in-stent neoatherosclerosis on long-term clinical outcomes: an observational study from the Kobe University Hospital optical coherence tomography registry. *EuroIntervention*. Dec 10.;12(11):e1366-e1374. 10.4244/EIJY15M12_05 10.4244/EIJY15M12_05 26690315

[11] Chen Z, Matsumura M, Mintz GS, Noguchi M, Fujimura T et al (2022) Prevalence and impact of neoatherosclerosis on clinical outcomes after percutaneous treatment of Second-Generation Drug-Eluting stent restenosis. Circ Cardiovasc Interv Sep 15(9):e011693. 10.1161/CIRCINTERVENTIONS.121.011693 10.1161/CIRCINTERVENTIONS.121.011693 36126137

[12] Shimono H, Kajiya T, Takaoka J, Miyamura A, Inoue T et al (2021) Characteristics of recurrent in-stent restenosis after second- and third-generation drug-eluting stent implantation. Coron Artery Dis Jan 32(1):36–41. 10.1097/MCA.0000000000000945 10.1097/MCA.0000000000000945 32826448

[13] Schwartz GG, Steg PG, Szarek M, Bhatt DL, Bittner VA et al (2018) Alirocumab and cardiovascular outcomes after acute coronary syndrome. N Engl J Med Nov 29(22):2097–2107. 10.1056/NEJMoa1801174 10.1056/NEJMoa1801174 30403574

[14] Raber L, Ueki Y, Otsuka T, Losdat S, Haner JD et al (2022) Effect of Alirocumab added to High-Intensity Statin therapy on coronary atherosclerosis in patients with acute myocardial infarction: the PACMAN-AMI randomized clinical trial. JAMA May 10(18):1771–1781. 10.1001/jama.2022.5218 10.1001/jama.2022.5218 PMC8978048 35368058

[15] Zanchin C, Koskinas KC, Ueki Y, Losdat S, Haner JD et al (2021) Effects of the PCSK9 antibody Alirocumab on coronary atherosclerosis in patients with acute myocardial infarction: a serial, multivessel, intravascular ultrasound, near-infrared spectroscopy and optical coherence tomography imaging study-Rationale and design of the PACMAN-AMI trial. Am Heart J Aug 238:33–44. 10.1016/j.ahj.2021.04.006 10.1016/j.ahj.2021.04.006 33951415

[16] Byrne RA, Rossello X, Coughlan JJ, Barbato E, Berry C et al (2023) 2023 ESC guidelines for the management of acute coronary syndromes. Eur Heart J Oct 12(38):3720–3826. 10.1093/eurheartj/ehad191 10.1093/eurheartj/ehad191 37622654

[17] Guagliumi G, Shimamura K, Sirbu V, Garbo R, Boccuzzi G et al (2018) Temporal course of vascular healing and neoatherosclerosis after implantation of durable- or biodegradable-polymer drug-eluting stents. Eur Heart J Jul 7(26):2448–2456. 10.1093/eurheartj/ehy273 10.1093/eurheartj/ehy273 29788263

[18] Tearney GJ, Regar E, Akasaka T, Adriaenssens T, Barlis P et al (2012) Consensus standards for acquisition, measurement, and reporting of intravascular optical coherence tomography studies: a report from the international working group for intravascular optical coherence tomography standardization and validation. J Am Coll Cardiol Mar 20(12):1058–1072. 10.1016/j.jacc.2011.09.079 10.1016/j.jacc.2011.09.079 22421299

[19] Hoshino M, Yonetsu T, Kanaji Y, Usui E, Yamaguchi M et al (2019) Impact of baseline plaque characteristic on the development of neoatherosclerosis in the very late phase after stenting. J Cardiol Jul 74(1):67–73. 10.1016/j.jjcc.2019.01.002 10.1016/j.jjcc.2019.01.002 30733110

[20] Nagano Y, Otake H, Toba T, Kuroda K, Shinkura Y et al (2019) Impaired Cholesterol-Uptake capacity of HDL might promote Target-Lesion revascularization by inducing neoatherosclerosis after stent implantation. J Am Heart Assoc May 7(9):e011975. 10.1161/JAHA.119.011975 10.1161/JAHA.119.011975 PMC6512103 30995875

[21] Kakizaki R, Häner J, Taniwaki M, Ohno Y, Yahagi K, THE IMPACT OF ACHIEVING GUIDELINE-ENDORSED LDL CHOLESTEROL LEVELS ON NEOATHEROSCLEROSIS FORMATION IN PATIENTS WITH STEMI (2025) : A SUBSTUDY OF THE RANDOMIZED CONNECT TRIAL. Presented at: American Control Conference 74th Annual Scientific Session; March ; Chicago, IL

[22] Niccoli G, Dato I, Imaeva AE, Antonazzo Panico R, Roberto M et al (2014) Association between inflammatory biomarkers and in-stent restenosis tissue features: an optical coherence tomography study. Eur Heart J Cardiovasc Imaging Aug 15(8):917–925. 10.1093/ehjci/jeu035 10.1093/ehjci/jeu035 24618655

[23] Cao YX, Li S, Liu HH, Li JJ (2018) Impact of PCSK9 monoclonal antibodies on Circulating hs-CRP levels: a systematic review and meta-analysis of randomised controlled trials. BMJ Open Oct 4(9):e022348. 10.1136/bmjopen-2018-022348 10.1136/bmjopen-2018-022348 PMC6173233 30287608

[24] Taniwaki M, Windecker S, Zaugg S, Stefanini GG, Baumgartner S et al (2015) The association between in-stent neoatherosclerosis and native coronary artery disease progression: a long-term angiographic and optical coherence tomography cohort study. Eur Heart J Aug 21(32):2167–2176. 10.1093/eurheartj/ehv227 10.1093/eurheartj/ehv227 26040806

[25] Sakai R, Sekimoto T, Koba S, Mori H, Matsukawa N et al (2023) Impact of triglyceride-rich lipoproteins on early in-stent neoatherosclerosis formation in patients undergoing Statin treatment. J Clin Lipidol Mar-Apr 17(2):281–290. 10.1016/j.jacl.2023.01.004 10.1016/j.jacl.2023.01.004 36828767

[26] Taniwaki M, Haner JD, Kakizaki R, Ohno Y, Yahagi K et al (2024) Long-term effect of biodegradable vs durable polymer everolimus-eluting stents on neoatherosclerosis in ST-segment elevation myocardial infarction: the CONNECT trial. Eur Heart J Sep 1. 10.1093/eurheartj/ehae589 10.1093/eurheartj/ehae589 39217617

[27] Jang JY, Kim JS, Shin DH, Kim BK, Ko YG et al (2015) Favorable effect of optimal lipid-lowering therapy on neointimal tissue characteristics after drug-eluting stent implantation: qualitative optical coherence tomographic analysis. Atherosclerosis Oct 242(2):553–559. 10.1016/j.atherosclerosis.2015.08.014 10.1016/j.atherosclerosis.2015.08.014 26318104

[28] Nakamura D, Dohi T, Ishihara T, Kikuchi A, Mori N et al (2021) Predictors and outcomes of neoatherosclerosis in patients with in-stent restenosis. EuroIntervention Aug 27(6):489–496. 10.4244/EIJ-D-20-00539 10.4244/EIJ-D-20-00539 PMC9725017 32985411

[29] Yuan X, Han Y, Hu X, Jiang M, Feng H et al (2023) Lipoprotein (a) is related to In-Stent neoatherosclerosis incidence rate and plaque vulnerability: optical coherence tomography study. Int J Cardiovasc Imaging Feb 39(2):275–284. 10.1007/s10554-022-02736-3 10.1007/s10554-022-02736-3 PMC9870965 36315364

[30] Furtado RHM, Fagundes AA Jr., Oyama K, Zelniker TA, Tang M et al (2022) Effect of Evolocumab in patients with prior percutaneous coronary intervention. Circ Cardiovasc Interv Mar 15(3):e011382. 10.1161/CIRCINTERVENTIONS.121.011382 10.1161/CIRCINTERVENTIONS.121.011382 35209731

[31] Yonetsu T, Kim JS, Kato K, Kim SJ, Xing L et al (2012) Comparison of incidence and time course of neoatherosclerosis between bare metal stents and drug-eluting stents using optical coherence tomography. Am J Cardiol Oct 1(7):933–939. 10.1016/j.amjcard.2012.05.027 10.1016/j.amjcard.2012.05.027 22727183

[32] Song L, Mintz GS, Yin D, Yamamoto MH, Chin CY et al (2017) Neoatherosclerosis assessed with optical coherence tomography in restenotic bare metal and first- and second-generation drug-eluting stents. Int J Cardiovasc Imaging Aug 33(8):1115–1124. 10.1007/s10554-017-1106-2 10.1007/s10554-017-1106-2 28281026

[33] Lee SY, Hur SH, Lee SG, Kim SW, Shin DH et al (2015) Optical coherence tomographic observation of in-stent neoatherosclerosis in lesions with more than 50% neointimal area stenosis after second-generation drug-eluting stent implantation. Circ Cardiovasc Interv Feb 8(2):e001878. 10.1161/CIRCINTERVENTIONS.114.001878 10.1161/CIRCINTERVENTIONS.114.001878 25613674

[34] Jinnouchi H, Otsuka F, Sato Y, Bhoite RR, Sakamoto A et al (2020) Healthy strut coverage after coronary stent implantation: an ex vivo human autopsy study. Circ Cardiovasc Interv May 13(5):e008869. 10.1161/CIRCINTERVENTIONS.119.008869 10.1161/CIRCINTERVENTIONS.119.008869 32338525

