# Supplementary material for: Impact of alirocumab on neoatherosclerosis formation and vessel healing after drug-eluting stent implantation in patients with acute myocardial infarction: a substudy of the PACMAN-AMI trial
Source: Int J Cardiovasc Imaging. 2025 Aug 18;41(9):1739–48. doi: 10.1007/s10554-025-03472-0 (PMC12405363; doi:10.1007/s10554-025-03472-0)
Supplement: Supplementary file 1 — Supplementary Material 1 [file 10554_2025_3472_MOESM1_ESM.docx]

**SUPPLEMENTARY MATERIALS**

**Impact of Alirocumab on Neoatherosclerosis Formation and Vessel Healing after Drug-eluting Stent Implantation in Patients with Acute Myocardial Infarction: a Substudy of the PACMAN-AMI Trial**

Ryota Kakizaki, Yasushi Ueki, Konstantinos C Koskinas, Hiroki Shibutani, Sylvain Losdat, Flavio G Biccirè, Tatsuhiko Otsuka, Jonas D Häner, Jacob Lønborg, Christoph Kaiser, Juan F Iglesias, Anna S Ondracek, David Spirk, George C M Siontis, Joost Daemen, Thomas Engstrøm, Irene M Lang, Lorenz Räber

**Supplementary tables 1-5**

**Supplementary figures 1-3**

**Supplementary Table 1.** Quantitative coronary angiography results comparing the alirocumab group vs. the placebo group

|  | **Alirocumab**  **n = 148** | **Placebo**  **n = 151** | **P value** |
| --- | --- | --- | --- |
| Post-PCI |  |  |  |
| Minimum lumen diameter, mm | 2.53 (0.51) | 2.57 (0.50) | 0.55 |
| Reference diameter, mm | 2.86 (0.55) | 2.90 (0.53) | 0.55 |
| % diameter stenosis, % | 11.34 (6.65) | 11.35 (5.69) | 0.99 |
| Follow-up |  |  |  |
| Minimum lumen diameter, mm | 2.30 (0.60) | 2.24 (0.66) | 0.46 |
| Reference diameter, mm | 2.72 (0.56) | 2.70 (0.55) | 0.82 |
| % diameter stenosis, % | 15.50 (13.41) | 17.47 (15.80) | 0.29 |
| Binary restenosis (≥50% diameter stenosis), n (%) | 5 (3.4%) | 6 (4.0%) | 1.00 |
| Late lumen loss, mm | 0.23 (0.39) | 0.33 (0.50) | 0.09 |

*Values are count (percentage) or mean (SD). P-values are from Student's t-tests or Fisher's exact test.*

**Supplementary Table 2.** Baseline patient, lesion, and procedural characteristics in patients with and without OCT imaging for culprit lesions at 1 year

|  | **Overall**  **n = 299** | **Patients with OCT**  **n = 191** | **Patients without OCT**  **n = 108** | **P value** | |
| --- | --- | --- | --- | --- | --- |
| **Patient characteristics** |  |  |  |  | |
| Age, years | 58.5 (9.7) | 58.1 (9.5) | 59.2 (10.0) | 0.38 | |
| Men, n (%) | 244 (81.3) | 160 (83.8) | 84 (77.1) | 0.17 | |
| Body mass index | 27.8 (4.3) | 27.9 (4.3) | 27.6 (4.4) | 0.62 | |
| Medical history, n (%) |  |  |  |  | |
| Arterial hypertension | 130 (43.3) | 81 (42.4) | 49 (45.0) | 0.72 | |
| Dyslipidemia | 238 (79.3) | 162 (84.8) | 76 (69.7) | 0.003 | |
| Diabetes | 31 (10.3) | 16 (8.4) | 15 (13.8) | 0.17 | |
| Current smoking | 142 (47.3) | 87 (45.5) | 55 (50.5) | 0.47 | |
| Previous myocardial infarction | 7 (2.3) | 2 (1.0) | 5 (4.6) | 0.10 | |
| Previous PCI | 7 (2.3) | 3 (1.6) | 4 (3.7) | 0.26 | |
| Peripheral arterial disease | 6 (2.0) | 3 (1.6) | 3 (2.8) | 0.67 | |
| Family history of CAD | 98 (32.7) | 59 (30.9) | 39 (35.8) | 0.44 | |
| Type of acute myocardial infarction, n (%) |  |  |  | 0.23 | |
| NSTEMI | 142 (47.3) | 85 (44.5) | 57 (52.3) |  | |
| STEMI | 158 (52.7) | 106 (55.5) | 52 (47.7) |  | |
| Peak CK, IU/L | 622 (967) | 687 (1061) | 505 (759) | 0.13 | |
| Peak hs-cTnT, ng/mL | 1132 (2804) | 1189 (2734) | 1026 (2939) | 0.64 | |
| LVEF, % | 53 (10) | 53 (11) | 53 (9) | 0.87 | |
| Medication, n (%) |  |  |  |  | |
| Statin | 37 (12.3) | 26 (13.6) | 11 (10.1) | 0.47 | |
| High-intensity statin therapy | 20 (6.7) | 13 (6.8) | 7 (6.4) | 1.00 | |
| Ezetimibe | 1 (0.3) | 0 (0.0) | 1 (0.9) | 0.36 | |
| Antiplatelet therapy |  |  |  |  | |
| Aspirin | 28 (9.3) | 13 (6.8) | 15 (13.8) | 0.06 | |
| P2Y12 inhibitor | 11 (3.7) | 3 (1.6) | 8 (7.3) | 0.020 | |
| Anti-coagulant | 6 (2.0) | 2 (1.0) | 4 (3.7) | 0.19 | |
| β-Blocker | 24 (8.0) | 13 (6.8) | 11 (10.1) | 0.38 | |
| ACEI | 41 (13.7) | 20 (10.5) | 21 (19.3) | 0.037 | |
| ARB | 29 (9.7) | 15 (7.9) | 14 (12.8) | 0.16 | |
| **Lesion and procedural characteristics** | | | | | |
| Target vessel location, n (%) |  |  |  | 0.14 | |
| Left anterior descending | 134 (44.8%) | 93 (48.7%) | 41 (38.0%) |  | |
| Left circumflex | 73 (24.4%) | 42 (22.0%) | 31 (28.7%) |  | |
| Right coronary artery | 91 (30.4%) | 56 (29.3%) | 35 (32.4%) |  | |
| Final TIMI flow |  |  |  | 0.30 | |
| 0 | 0 (0.0%) | 0 (0.0%) | 0 (0.0%) |  | |
| 1 | 0 (0.0%) | 0 (0.0%) | 0 (0.0%) |  | |
| 2 | 3 (1.0%) | 1 (0.5%) | 2 (1.9%) |  | |
| 3 | 296 (99.0%) | 190 (99.5%) | 106 (98.1%) |  | |
| Number of stents | 1.0 [0.0] | 1.0 [0.0] | 1.0 [0.0] | 0.73 | |
| Total stent length, mm | 26.0 [20.0] | 28.0 [20.0] | 26.0 [20.0] | 0.45 | |
| Stent diameter, mm | 3.5 [1.0] | 3.5 [1.0] | 3.5 [1.5] | 0.33 | |
| Post balloon dilatation, n (%) | 227 (75.9%) | 160 (83.8%) | 67 (62.0%) | <0.001 | |
| Polymer type, n (%) |  |  |  |  | |
| Biodegradable polymer | 126 (42.1%) | 69 (36.1%) | 57 (52.8%) |  | |
| Durable polymer | 161 (53.8%) | 118 (61.8%) | 43 (39.8%) | 0.001 | |
| Bifurcation lesion, n (%) | 40 (13.4%) | 30 (15.7%) | 10 (9.3%) | 0.16 | |
| Multivessel PCI, n (%) | 29 (9.7%) | 19 (9.9%) | 10 (9.3%) | 1.00 | |
| GP IIb/IIIa inhibitors, n (%) | 40 (13.4%) | 33 (17.3%) | 7 (6.5%) | 0.008 | |
| **Quantitative coronary angiography analysis** | | | | | |
| Post-PCI |  |  |  |  | |
| Minimum lumen diameter, mm | 2.55 (0.50) | 2.55 (0.47) | 2.57 (0.61) | 0.81 | |
| Reference diameter, mm | 2.88 (0.54) | 2.88 (0.51) | 2.90 (0.62) | 0.78 | |
| % diameter stenosis, % | 11.34 (6.16) | 11.21 (6.34) | 11.72 (5.64) | 0.57 | |
| Follow-up |  |  |  |  | |
| Minimum lumen diameter, mm | 2.27 (0.63) | 2.26 (0.61) | 2.33 (0.70) | 0.44 | |
| Reference diameter, mm | 2.71 (0.55) | 2.69 (0.52) | 2.79 (0.63) | 0.22 | |
| % diameter stenosis, % | 16.51 (14.68) | 16.23 (14.92) | 17.34 (14.04) | 0.60 | |
| Binary restenosis (≥50% diameter stenosis), n (%) | 11 (3.7%) | 8 (4.2%) | 3 (2.8%) | 1.00 | |
| Late lumen loss, mm | 0.28 (0.45) | 0.29 (0.46) | 0.24 (0.41) | 0.42 | |
| *Values are count (percentage), mean (SD), or median [interquartile range]. P-values are from Student’s t-tests, Fisher’s exact tests or Wilcoxon-Mann-Whitney tests. Abbreviations: ACEI: angiotensin converting enzyme inhibitor, ARB: angiotensin receptor blocker, BMI: body mass index, CAD: coronary artery disease, CK: creatine kinase, cTnT: cardiac troponin T, GP: glycoprotein, LVEF: left ventricular ejection fraction, NSTEMI: non-ST-segment elevation myocardial infarction, PCI: percutaneous coronary intervention, STEMI: ST-segment elevation myocardial infarction, TIMI: thrombolysis in myocardial infarction* | | | | |  |

**Supplementary Table 3.** List of patients with neoatherosclerosis

| **No.** | **Group** | **Age** | **Vessel** | **Sex** | **DM** | **Smoking** | **Dx** | **In-stent**  **fibroatheroma** | **In-stent**  **fibrocalcific plaque** | **In-stent**  **macrophage** | **In-stent**  **Cholesterol crystal** | **LDL-C** | | **hsCRP** | |
| --- | --- | --- | --- | --- | --- | --- | --- | --- | --- | --- | --- | --- | --- | --- | --- |
|  |  |  |  |  |  |  |  |  |  |  |  | **Baseline** | **Week 52** | **Baseline** | **Week 52** |
| 1 | Alirocumab | 64 | LAD | Male | No | yes | STEMI | Yes | No | Yes | Yes | 164.09 | 24.32 | 1.40 | 2.80 |
| 2 | Alirocumab | 57 | RCA | Male | No | yes | NSTEMI | Yes | No | No | No | 178.76 | 9.27 | 2.00 | 0.00 |
| 3 | Alirocumab | 56 | LCx | Male | No | no | STEMI | Yes | No | No | No | 158.30 | 17.76 | 1.00 | 0.00 |
| 4 | Alirocumab | 60 | LAD | Male | Yes | yes | NSTEMI | Yes | No | No | No | 192.28 | 3.86 | 4.10 | 0.80 |
| 5 | Placebo | 53 | RCA | Male | No | yes | NSTEMI | Yes | No | No | Yes | 115.06 | 44.02 | 1.10 | 0.00 |
| 6 | Placebo | 72 | RCA | Female | Yes | no | STEMI | Yes | No | Yes | No | 211.58 | 60.23 | 9.70 | 0.00 |
| 7 | Placebo | 56 | RCA | Male | No | yes | STEMI | Yes | No | No | No | 141.31 | 49.81 | 10.10 | 0.80 |
| 8 | Placebo | 61 | RCA | Male | No | no | STEMI | Yes | Yes | No | No | 125.10 | 48.65 | 2.70 | 2.20 |
| 9 | Placebo | 41 | RCA | Male | No | yes | NSTEMI | Yes | No | No | No | 130.50 | 76.45 | 9.90 | 1.50 |
| 10 | Placebo | 54 | LAD | Male | No | no | NSTEMI | Yes | No | No | No | 156.37 | 61.39 | 33.80 | 2.30 |
| 11 | Placebo | 54 | RCA | Male | No | no | STEMI | Yes | Yes | No | No | 152.12 | 66.02 | 0.90 | 0.00 |
| 12 | Placebo | 62 | Diagonal | Male | No | no | NSTEMI | No | No | No | Yes | 100.00 | 76.06 | 0.00 | 0.00 |
| 13 | Placebo | 60 | RCA | Male | No | yes | STEMI | No | No | No | Yes | 167.95 | 71.43 | 5.40 | 1.90 |
| *Values are raw (LDL-C) or mean per patient across vessels (IVUS, NIRS, OCT). Abbreviations: DM: diabetes mellitus, Dx: diagnosis, FCT: fibrous cap thickness, hsCRP: high sensitive C-reactive protein, IRA: infarct-related arteries, IVUS: intravascular ultrasound, LAD: left anterior descending, LCBI: lipid core burden index, LCx: Left circumflex, LDL-C: low density lipoprotein cholesterol, OCT: optical coherence tomography, RCA: Right coronary artery* | | | | | | | | | | | | | | | |

**Supplementary Table 4.** Biochemical measures and intracoronary imaging findings in patients with and without neoatherosclerosis

|  | **Neoatherosclerosis (+)**  **N = 13** | **Neoatherosclerosis (-)**  **N = 178** |  |
| --- | --- | --- | --- |
| **Biochemical measures** |  |  |  |
| Total cholesterol, mg/dL |  |  |  |
| Baseline | 203.1 (29.6) | 206.4 (33.2) |  |
| 1 year | 108.4 (33.9) | 112.1 (41.4) |  |
| Absolute change | -94.7 (-125.3 to -64.2) | -94.3 (-101.4 to -87.3) |  |
| LDL- cholesterol, mg/dL |  |  |  |
| Baseline | 153.3 (31.2) | 153.4 (33.5) |  |
| 1 year | 46.9 (25.4) | 49.1 (37.9) |  |
| Absolute change | -106.5 (-135.4 to -77.5) | -104.3 (-111.0 to -97.6) |  |
| HDL- cholesterol, mg/dL |  |  |  |
| Baseline | 37.6 (6.7) | 42.3 (9.9) |  |
| 1 year | 45.1 (10.1) | 47.6 (11.5) |  |
| Absolute change | 7.5 (3.1 to 12.0) | 5.4 (4.3 to 6.5) |  |
| Non-HDL- cholesterol, mg/dL |  |  |  |
| Baseline | 165.7 (32.7) | 164.4 (33.9) |  |
| 1 year | 63.3 (32.2) | 64.6 (42.2) |  |
| Absolute change | -102.5 (-133.3 to -71.6) | -99.8 (-106.9 to -92.7) |  |
| Triglycerides, mg/dL |  |  |  |
| Baseline | 124.4 (81.1) | 105.5 (77.3) |  |
| 1 year | 122.1 (56.2) | 103.6 (61.2) |  |
| Absolute change | -2.3 (-33.2 to 28.6) | -1.9 (-10.9 to 7.2) |  |
| Lipoprotein(a), mg/dL |  |  |  |
| Baseline | 29.9 (39.3) | 31.1 (39.8) |  |
| 1 year | 36.4 (46.6) | 33.5 (45.5) |  |
| Absolute change | 6.6 (-3.3 to 16.4) | 2.4 (0.3 to 4.6) |  |
| Apolipoprotein AI, mg/dL |  |  |  |
| Baseline | 108.5 (13.2) | 115.1 (18.7) |  |
| 1 year | 130.0 (19.3) | 130.8 (20.7) |  |
| Absolute change | 21.5 (12.0 to 30.9) | 15.7 (13.4 to 18.0) |  |
| Apolipoprotein B, mg/dL |  |  |  |
| Baseline | 116.2 (21.5) | 114.1 (22.0) |  |
| 1 year | 52.5 (21.5) | 51.8 (28.6) |  |
| Absolute change | -63.7 (-84.1 to -43.3) | -62.2 (-66.9 to -57.6) |  |
| High-sensitivity CRP, mg/L |  |  |  |
| Baseline | 6.3 (9.0) | 6.0 (11.8) |  |
| 1 year | 0.9 (1.1) | 2.4 (4.3) |  |
| Absolute change | -5.4 (-10.6 to -0.1) | -3.6 (-5.4 to -1.8) |  |
| **Intracoronary imaging findings** |  |  |  |
| Non-IRA PAV in IVUS |  |  |  |
| Baseline | 42.9 (6.4) | 42.0 (8.0) |  |
| 1 year | 41.3 (6.8) | 40.4 (8.2) |  |
| Absolute change | -1.6 (-3.0 to -0.3) | -1.7 (-2.0 to -1.3) |  |
| Non-IRA max LCBI 4mm in NIRS |  |  |  |
| Baseline | 379.4 (191.4) | 262.9 (147.2) |  |
| 1 year | 331.0 (186.6) | 207.2 (160.4) |  |
| Absolute change | -48.4 (-108.1 to 11.2) | -55.7 (-75.3 to -36.0) |  |
| Non-IRA minimum FCT in OCT |  |  |  |
| Baseline | 78.0 (24.8) | 108.8 (60.5) |  |
| 1 year | 128.9 (78.6) | 153.8 (79.2) |  |
| Absolute change | 50.9 (13.0 to 88.8) | 45.1 (33.8 to 56.3) |  |
| *Values are mean (SD) or mean (95% CI). Abbreviations: CRP: C-reactive protein, eGFR: estimated glomerular filtration rate, FCT: fibrous cap thickness, HbA1c: hemoglobin A1c, HDL: high density lipoprotein, IRA: infarct-related arteries, IVUS: intravascular ultrasound, LCBI: lipid core burden index, LDL: low density lipoprotein, OCT: optical coherence tomography* | | | |

**Supplementary Table 5.** OCT parameters in patients with and without neoatherosclerosis

|  | **Neoatherosclerosis (+)**  **N = 13** | **Neoatherosclerosis (-)**  **N = 178** |
| --- | --- | --- |
| Duration from Implantation, day | 369 (11) | 376 (15) |
| Analysed cross section per lesion, n | 107 (39) | 76 (34) |
| Analysed stent length, mm | 42.9 (15.4) | 30.2 (13.7) |
| Minimal lumen area, mm2 | 3.41 (1.83) | 4.92 (2.21) |
| Minimal stent area, mm2 | 6.26 (3.14) | 6.04 (2.40) |
| Neointimal area, mm2 | 2.11 (1.08) | 1.06 (0.79) |
| Rate of cross section with mean neointimal thickness>100, % | 79.4 (21.0) | 56.3 (33.2) |
| Rate of cross section with any uncovered struts, % | 4.3 (6.0) | 14.3 (16.3) |
| Rate of cross section with any malapposed struts, % | 3.7 (10.1) | 2.7 (7.6) |
| Analysed struts per lesion, n (%) | 921 (362) | 751 (362) |
| Mean neointimal thickness, μm | 241.41 (96.72) | 136.70 (82.96) |
| Rate of uncovered struts, % | 0.75 (1.04) | 2.84 (4.00) |
| Rate of malapposed struts, % | 1.79 (5.06) | 0.66 (2.13) |
| *Values are mean (SD) or count (%). Abbreviations: OCT: optical coherence tomography* | | |

**Supplementary Table 6.** Clinical outcomes

|  | **Overall**  **n = 191** | **Neoatherosclerosis (+)**  **N = 13** | **Neoatherosclerosis (-)**  **N = 178** | |
| --- | --- | --- | --- | --- |
| Any revascularization | 35 (18.3%) | 3 (23.1%) | 32 (18.0%) | |
| Ischemia driven revascularization | 30 (15.7%) | 3 (23.1%) | 27 (15.2%) | |
| Ischemia driven target lesion revascularization | 11 (5.8%) | 2 (15.4%) | 9 (5.1%) | |
| Ischemia driven non-target lesion revascularization | 19 (9.9%) | 1 (7.7%) | 18 (10.1%) | |
| *Values are count (percentage).* | | | |  |

**Supplementary Table 7.** Stent type

|  | **Overall**  **n = 187** | **Alirocumab**  **n = 92** | **Placebo**  **n = 95** |
| --- | --- | --- | --- |
| Xience (Abbott) | 51 (26.7%) | 24 (25.3%) | 27 (28.1%) |
| Resolute (Medtronic) | 38 (19.9%) | 19 (20.0%) | 19 (19.8%) |
| Orsiro (BIOTRONIK) | 39 (20.4%) | 21 (22.1%) | 18 (18.8%) |
| Synergy (Boston Scientific) | 25 (13.1%) | 12 (12.6%) | 13 (13.5%) |
| Ultimaster (Terumo) | 29 (15.2%) | 14 (14.7%) | 15 (15.6%) |
| Biomatrix (Biosensors) | 4 (2.1%) | 2 (2.1%) | 2 (2.1%) |
| Firehawk (MicroPort) | 1 (0.5%) | 0 (0.0%) | 1 (1.0%) |
| Values are count (percentage). | | | |

**Supplementary Figure 1.** Study flow chart


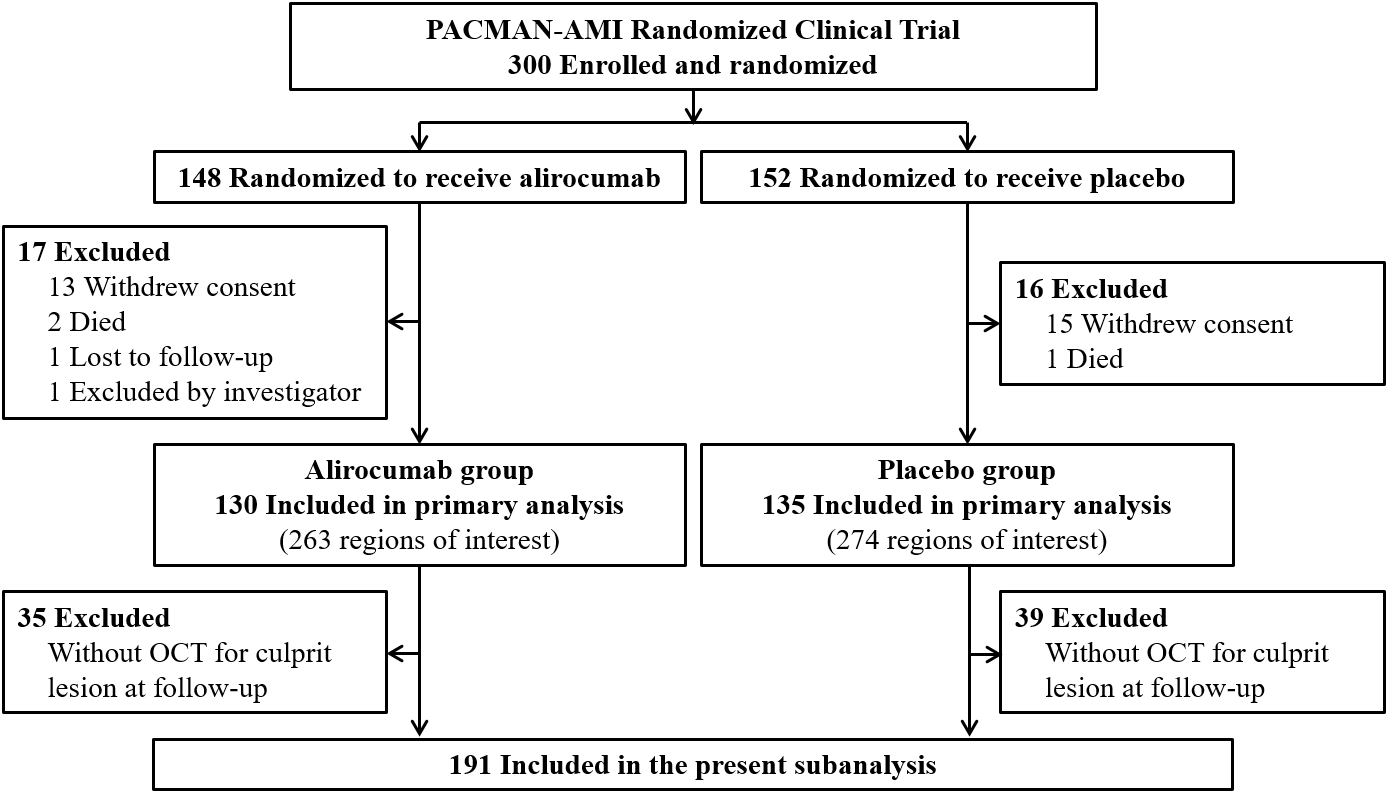


OCT: optical coherence tomography

**Supplementary Figure 2.** Correlation between neointimal area and absolute change of LDL-C


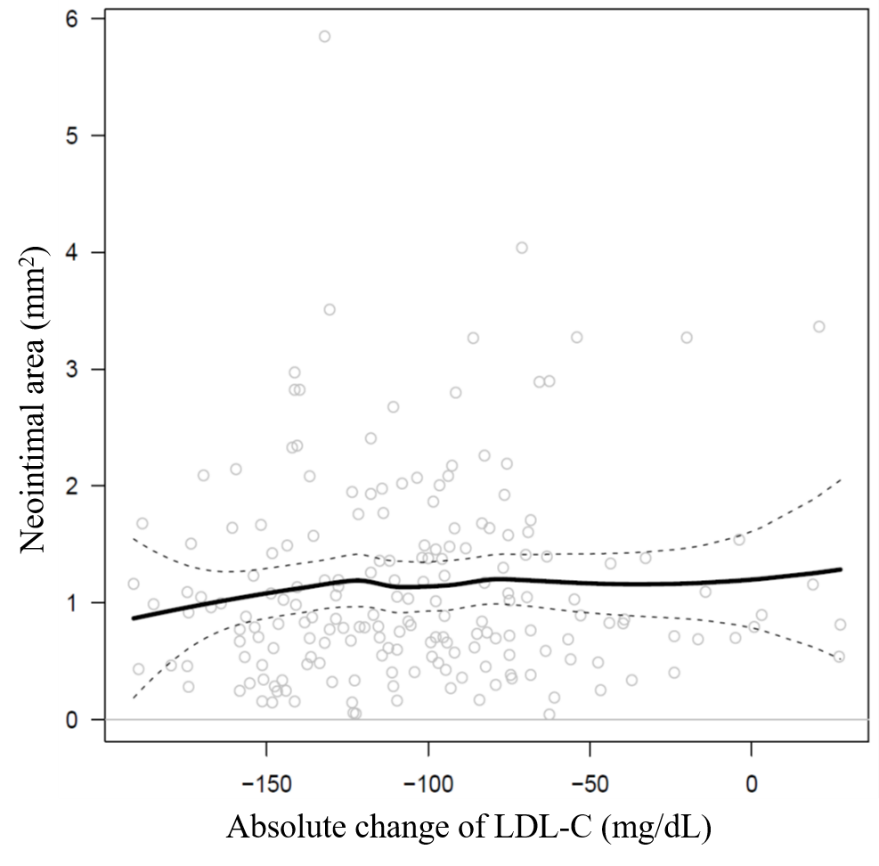


LDL-C: low density lipoprotein cholesterol

**Supplementary Figure 3.** Correlation between percentage of uncovered struts and absolute change of LDL-C

**
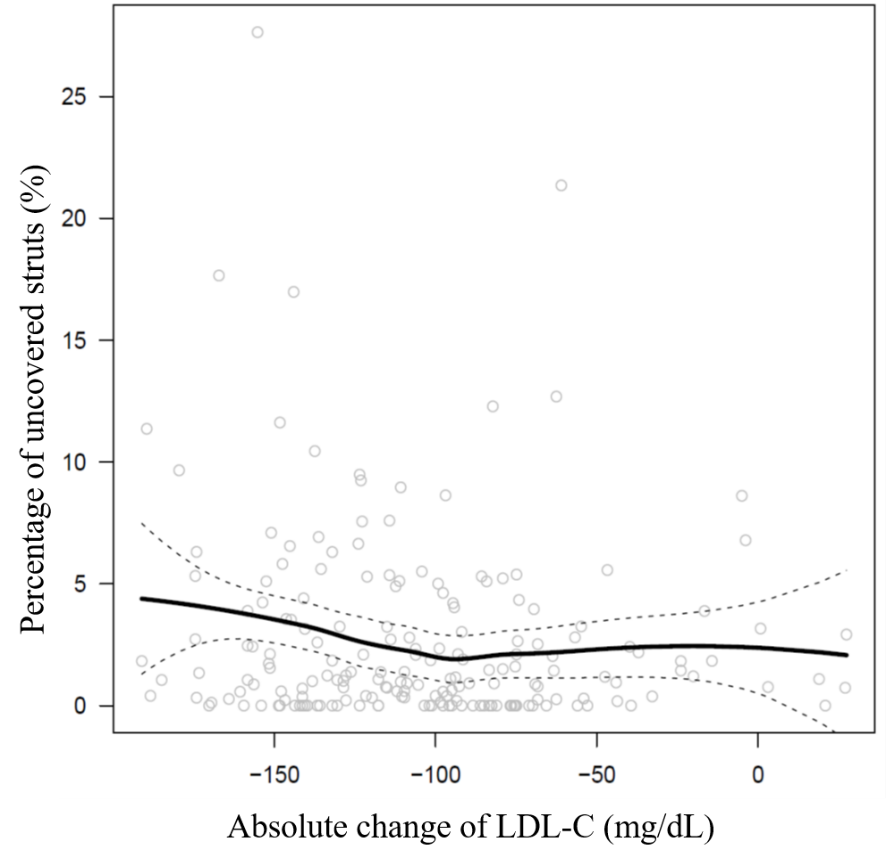
**

LDL-C: low density lipoprotein cholesterol
